# The relationship between job stress and patient safety culture among nurses: a systematic review

**DOI:** 10.1186/s12912-023-01198-9

**Published:** 2023-02-13

**Authors:** Loai M. Zabin, Rasha S. Abu Zaitoun, Esa M. Sweity, Lila de Tantillo

**Affiliations:** 1 Department of Nursing, An-Najah National University Hospital, Nablus, 44839 Palestine; 2 Keigwin School of Nursing, Jacksonville University, 2800 University Blvd. North, Jacksonville, FL 32221 USA

**Keywords:** Patient safety, Safety culture, Occupational stress, Job stress, Nurses

## Abstract

**Background:**

Work stress is one of the leading causes of physical and mental problems among nurses and can affect patient safety. Nurses experiencing stress are more prone to make errors, which has consequences for the safety culture. This study aimed to describe the findings of studies that examined the relationship between job stress and patient safety culture among nurses.

**Methods:**

A systematic review of published English-language articles from 2017 to 2021 was obtained through an electronic search of three large online databases (i.e., CINAHL through EBSCOhost, Medline through PubMed, and Embase). We used the Statement of Preferred Reporting Items for Systematic Reviews and Meta-Analyses (PRISMA) to guide the undertaking of this review. In addition, data extraction and quality assessment were performed for the final seven quantitative articles.

**Results:**

This review showed a significant relationship between job-related stress in its different factors, patient safety culture, and patient safety. Three studies of the seven reviewed articles examined the relationship. The rest of the studies examined the relationship indirectly, discussing factors that impacted job stress and how they affected patient safety culture. However, differences in working conditions and study characteristics affected the results of these studies and the significance of this relationship.

**Conclusions:**

This review suggests that nursing managers and administrators should consider actions to minimize nursing job stress to the minimum levels and improve their work environment to provide the best possible patient care. Future studies are needed to develop interventions to reduce workplace stress and improve nurses' safety. Furthermore, nurses’ managers and educators should train nurses on resilience and how to work in trauma-informed care.

**Supplementary Information:**

The online version contains supplementary material available at 10.1186/s12912-023-01198-9.

## Background

The level of healthcare quality is critically dependent on patient safety. To consistently improve the level of care, it is becoming more widely acknowledged that health organizations need to improve their safety cultures [1]. Patient safety has been viewed as one of the key elements of healthcare administration [2]. According to Kohn et al. [3], safety is an essential and fundamental component of research on patient care. Safety culture is the combination of individual and group beliefs, attitudes, competencies, and behavioral patterns that shape an organization's commitment to attitude and level of competency in managing the safety of patients [1, 4]. The Agency for Healthcare Research and Quality (AHRQ) also defines patient safety culture as the degree to which an organization's culture supports and promotes patient safety [5].

The health-work interface has been recognized as a topic that must be discussed in today's world, particularly in the hospital setting when it comes to patient safety. Workers' health and working conditions appear to be crucial factors to consider while seeking safe treatment [6].

Job stress is one of the leading reasons for physical and mental problems among healthcare staff and lower productivity in healthcare organizations. It may affect the quality of health services, especially among nurses [7, 8]. Job stress is a harmful physical and emotional response that can occur when an employee is confronted with job demands and pressures that are out of relation to their knowledge, capabilities, and abilities, making it difficult to cope with [9]. Nursing is a stressful and high-risk profession, and nurses face job stress at work with great frequency [10, 11]. In many nations, nurses account for 50% of all health care professionals. They play a critical role in the management and front-line implementation of health measures [12]. While personally caring for patients, nurses play a crucial role in maintaining patient safety, since they spend much of their time with patients [13]. However, shortage of staff, work pressure, under-reporting of adverse events, and professionals' lack of continuing education all have an impact on the emergence of stress and adverse events, which helps to foster an environment where mistakes can happen and patients can suffer harm [14].

The impact of health injuries from stress on healthcare personnel can jeopardize patient safety. The exhausted health care staff and their difficulty coping with job stress may negatively impact the safety culture [6]. Job-related stress affects organizational performance, staff, and service outcomes [15]. Nurses are responsible for maintaining patient safety while providing health care. Thus, following safety standards while serving everyday treatment could reduce adverse events and damages [7]. Previous studies have indicated a positive relationship between organizational learning, good teamwork, and communication about errors as factors for improving patient safety culture [16–18]. However, few studies have examined the relationship between job stress and patient safety culture. To our knowledge, no systematic review has been conducted to address this relationship. Therefore, this literature review aims to find and analyze the studies to increase the available knowledge regarding the relationship between job stress and patient safety culture.

## Methods

### Study design

A systematic review of the published literature was conducted from March 29, 2022, through April 10, 2022. We collected data through an electronic search for the last five years (from 2017 through 2022) to ensure that we examined the most recent studies.

### Search strategy

**Fig. 1:**
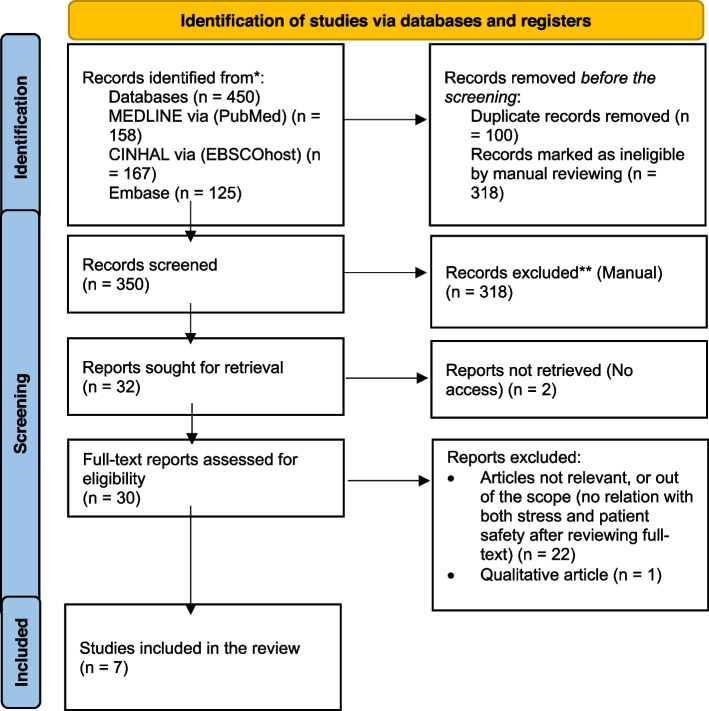
PRISMA flow diagram of the systematic review

We used the following databases to choose the articles: MEDLINE (via PubMed), CINHAL (via EBSCOhost), and Embase. The search approach employed the Boolean operator OR between keywords *nursing*, safety, and *stress* AND comparable MeSH phrases (see Table 1). To refine the search phrases with diverse meanings were joined using the Boolean operator AND. The search approach utilized for PubMed-MEDLINE is described in Additional File 1. The method was the same for the other databases, but it was tailored to each one's unique characteristics. We documented the search methods and findings following the relevant sections of the Preferred Reporting Items for Systematic Reviews and Meta-Analyses (PRISMA) statement [19] (Fig. 1). Then the abstracts of all identified papers were screened to determine their relevance to this study.

**Table 1:** Search strategy

Term connected by OR	AND	Term connected by OR	AND	Term connected by OR
Occupational Stress – MeSH: **OR**		Patient safety culture **OR**		“Nursing” **OR**
Job Stress		Safety Culture		Nurses
work stress		Patient safety		Nursing Staff
Job related stress		Safety climate		Registered Nurses
Professional Stress				Nursing Personnel
Organizational stress				Nurse*
Workplace Stress				
Work related stress				

*: it indicates that the search equation does not include other terms after "nurse". this is to ensure that the search only includes nurses

Given the objective of this review of the literature to examine the relationship between job stress and patient safety culture among nurses, we decided to choose articles that met the following criteria: (1) studies using the quantitative approach; (2) studies conducted with nurse participants; (3) articles in the English language; (4) stress was related to the job of nurses. Conversely, the following studies were excluded: (1) those in the form of letters/opinions, editorials, essays, case studies and literature, comments or narrative, and systematic reviews; (2) articles with no abstracts and full text; (3) articles published in other languages than English; (4) studies with no nurse participants; (5) studies used qualitative approach; (6) articles without access.

### Data extraction and management

Our search in the three databases (PubMed, CINHAL via EBSCOhost, and Embase) resulted in 450 articles. After removing the duplicated titles, 350 were screened for relevance. Thirty-two articles were sought for retrieval; however, we could not access two of them. We excluded one article that uses the qualitative approach because it is out of our inclusion criteria. Twenty-two articles were also excluded after reviewing the abstracts, as they are outside of the scope of our review (they did not discuss the relationship between job stress and patient safety culture). Finally, we reviewed the full text of seven articles and found that they have good relevance to our systematic review goal [7, 20–25].

### Risk of bias assessment

This study used the JBI Critical Appraisal Checklist for Systematic Reviews to assess the methodological quality of a study and to address the possibility of bias [26]. Two reviewers (LZ and RZ) utilized the tool to evaluate each article meeting inclusion criteria. Disputes in assessing the risk of bias were handled by debate and consensus between the two authors, with any persistent disagreements referred to a third author (ES). All seven articles were determined to meet quality standards and were included in the review.

## Results

Our search resulted in seven related articles. We reviewed the full text of these articles to look for the relationship between job stress and patient safety culture among nurses. The reviewed articles were all cross-sectional studies. However, one study used a cross-sectional, multicenter, mixed-methods approach [25], and one used descriptive correlational [22]. The size of the samples ranged from 143 [24] to 1671 [21], with a response rate ranging from 54.4% [22] to 92% [7]. Most of the studies were conducted in Iran [7, 20, 24], and other studies were conducted in Turkey [22], China [21], Oman [23], and Germany [25]. Detailed characteristics of the studies can be viewed in Table 2.

**Table 2:** Properties of the studies reviewed

Author / Publication year	Region	Design	Population	Sample size	Response rate	Tools	Quality
**M Poursadeqiyan, MF Arefi, S Khaleghi, AS Moghaddam, E Mazloumi, M Raei, M Hami and A Khammar ** [24]	Iran	Descriptive-analytical and cross-sectional	Nurses	143	Not mentioned	SOFI NSC	Low
**S Asefzadeh, R Kalhor and M Tir ** [7]	Iran	Cross-sectional	Nurses	380	92	HSOPSC SSJSQ	High
**MS Keykaleh, H Safarpour, S Yousefian, F Faghisolouk, E Mohammadi and Z Ghomian ** [20]	Iran	Cross-sectional	Nurses and patients	200	Not mentioned	Self-developed	Good
**J Liu, J Zheng, K Liu, X Liu, Y Wu, J Wang and L You ** [21]	China	Cross-sectional	Nurses	1671	89.9	CNS HSOPSC	Good
**Q Al Ma'mari, LA Sharour and O Al Omari ** [23]	Oman	Cross-sectional	Nurses	300	90	PES-NWI HSOPSC	Good
**G Yalcin Akgul and N Aksoy ** [22]	Turkey	Descriptive correlational	OR medical staff including nurses	164	54.4	OSS SAQ-OR	Good
**H Sturm, MA Rieger, P Martus, E Ueding, A Wagner, M Holderried, J Maschmann and C WorkSafeMed ** [25]	Germany	Cross-sectional, multicenter, mixed-methods	Physicians and Nurses	1502	76	COPSOQ HSPSC-D	Good

*SOFI* Swedish Occupational Fatigue Inventory, *NSC* Nurses Safety Climate, *HSOPSC* Hospital Survey on Patient Safety Culture, *SSJSQ* Stinemetz Standard Job Stress Questionnaire, *CNS* China Nurse Survey, *PES-NWI* Practice Environment Scale of the Nursing Work Index, *OSS* Organizational Stress Scale, *SAQ-OR* Safety Attitudes Questionnaire—Operating Room, *COPSOQ* Copenhagen Psychosocial Questionnaire

Regarding the tools used to assess job stress among nurses, study researchers used different instruments, one of which was self-developed [20]. However, most studies used the Hospital Survey of Patient Safety Culture (HSOPSC) to assess the level of safety culture among nurses more frequently than any other [7, 21, 23, 25]. In addition, the Safety Attitudes Questionnaire (SAQ) was used in one study [22], the Nurses Safety Climate Assessment Questionnaire was also used in one study [24], and a self-developed questionnaire using a patient safety checklist with 44 items was used in the last study [20].

### Study objectives

Two studies were conducted to examine directly job stress and patient safety culture [7, 22]. These studies presented the need to identify and reduce nursing stressors to improve patient safety and safety culture. The remaining studies had goals very similar to the previous two studies, but they did not directly use a tool to measure job stress. For example, Keykaleh et al. [20] examined job stress and its relationship with patient safety culture with a self-developed checklist focused on the safety of patients, not the perceived cultures of nursing staff. In the same manner, Liu et al. [21] and Al Ma'mari et al. [23] did the same but did not measure the job stress of nurses directly. They examined workplace violence and burnout as a result of job stress and workload on the safety of patients as perceived by nurses. They posited that workplace violence, burnout, and workload would lead to more stress, leading to more adverse events and therefore affecting the safety of patients. Furthermore, Poursadeqiyan et al. [24] examined the relationship between occupational fatigue – which resulted in cumulative stress and burnout – and the safety climate. Sturm et al. [25] used a mixed-method study to examine the relationship between safety culture and patient safety with work-related stress and work conditions.

### Job stress and patient safety culture

The relationship between job stress and safety culture fluctuated in the different studies. For example, the Pearson’s correlation test by Asefzadeh et al. [7] showed a significant relationship between some dimensions of patient safety culture and work stress levels (*p* ≤ 0.05). However, the Spearman correlation test run by Keykaleh et al. [20] did not show a significant relationship between patient safety culture and job stress (*r* = 0.007 and *p* = 0.919). However, he showed some factors that have a great stress-related impact on nurses, including long working hours, working on holidays, high workload, lack of career development, lack of right thinking towards nursing professions at the societal level, and the unappreciated value of nursing careers for others. These factors may increase nursing errors and adverse events. Yalcin Akgul and Aksoy [22] have found a negative and weak correlation between safety attitude and organizational stress as perceived by medical staff working in OR.

### Indirect relationship

The rest of the studies showed different relations between patient safety culture with various sources of job stress (e.g., workload, burnout, working conditions, workplace violence, and fatigue). For example, Liu et al. [21] showed that workplace violence was associated with a higher average of burnout and adverse events, putting patients' safety at risk. Al Ma'mari et al. [23] found that workload does not significantly correlate with the overall perception of safety. However, fatigue, emotional exhaustion, and depersonalization have a detrimental effect on nurses’ perceptions of safety. Furthermore, Poursadeqiyan et al. [24] have found that occupational fatigue and burnout negatively affected the safety climate. Sturm et al. [25] also found that workload correlated directly with work-related stress and patient safety outcomes, as perceived by nurses.

## Discussion

In this systematic review, 350 journal articles were screened, and only 29 selected articles contained quantitative information on the relationship between job stress or factors associated with job stress and patient safety culture within the nursing field. However, not all of these articles directly examined the relationship between job stress and patient safety culture. Only three articles discussed the relationship directly, which made it difficult to focus on this exact relationship. The rest of the related articles were four, and they examined the relationship between patient safety culture and factors related to job stress, such as fatigue, workload, workplace violence, and burnout.

Three articles discussed the relationship between occupational stress or job stress and patient safety culture. The findings of these studies showed that a significant negative relationship was found between job stress and patient safety culture [7, 22, 24]. However, it was not within the same power in all studies. For example, Yalcin Akgul and Aksoy [22] explained that the relationship was weak. That could be because the study was conducted in one unit with a small number of nurses participating. Other studies showed that many factors such as fatigue, workload, burnout, and workplace violence impacted staff’s job stress and lowered patient safety culture [21, 23, 25]. That is because these factors could increase nurses' stress, making them more prone to mistakes and adverse events, which in turn has been associated with patient safety. Although Keykaleh et al. [20] have found factors that greatly impacted nurses' stress, no significant relationship between nurses' job stress and patient safety culture was found. That could be due to the differences in working conditions in the hospitals where this study was conducted.

It is wise to note that this review was based on the findings of seven studies with different results. There are many possible causes for the differences in the findings. The first might be the sample size and study population disparity. For example, in one study, the population was all OR staff including nurses, physicians, and technicians [22]. Another study focused on nurses and physicians [25]. However, other studies were conducted exclusively on nurses [7, 20, 21, 23, 24]. The second reason for the disparity could be differences in socioeconomic status and measurement instruments. For example, Sturm et al. [25] used the German version of the Hospital Survey on Patient Safety Culture, which had some differences in the number of components compared to other versions of the same tool. Other studies used different tools to measure patient safety culture, which had some variations in the components compared to other tools [20–22, 24]. That also applies to measuring job stress, as different tools with variations in their components were used. Using different tools means that some studies assessed the relationship between job stress and some dimensions of patient safety culture with different approaches. For instance, Al Ma'mari et al. [23] assessed the relationship between job stress variables with an overall perception of the patient safety culture dimension. However, Asefzadeh et al. [7] assessed the relationship between job stress with each dimension of patient safety culture separately. In the rest of the studies reviewed, the lack of consistency among tools made it difficult to evaluate specific dimensions of other tools in comparison to others.

Another reason for the differences in the relationship found in this review might be the different working conditions for nurses, affecting job stress. For example, the type of hospitals (educational, private, or government), the number of patients, the number of staff, financial means [22], and other related conditions can affect the overall results of job-related stress and also the perception of the patient safety culture [20]. In addition, nurses' demographic characteristics could affect the overall level of stress at work. However, not all have a negative relationship, due to the differences between their living conditions and their work conditions [7, 20].

To our knowledge, this is the first conducted systematic review that investigates the relationship between job stress and patient safety culture. However, the studies included in this review have some limitations. The first limitation was that all of them were cross-sectional. While all articles in the review to examined the impact of job stress on patient safety culture, cross-sectional investigations do not exclude the possibility of a bidirectional relationship between the variables. The possibility of influence from patient safety culture on job stress merits future investigation. In addition, most were conducted at a single site or in a single unit in a hospital, limiting the generalizability of results. The next limitation was that this review did not include the gray literature. Finally, the last limitation could be attributed to the heterogeneity of the studies, which the readers should consider when applying this conclusion.

## Conclusions

The finding of this review suggests that there is a negative relationship between job stress and patient safety culture. The cornerstone of promoting patient safety and quality of treatment in healthcare institutions is the patient safety culture. Building a strong patient safety culture is critical for healthcare institutions to promote patient safety while improving the quality of care. Job stress seems to be one of the barriers to improving the patient safety culture, which requires the development of effective stress management measures for nurses and other healthcare staff. Therefore, administrators and nursing managers should consider nursing stress in the workplace a safety issue. More research is needed to develop interventions to reduce nursing stress in the workplace and improve their work environment and provide safe patient care at an optimal level.

### Recommendation and implications for nursing

The findings of this study suggest more work is needed to find strategies to reduce workplace stress and support nurses’ safety at work. Future research is also recommended to explore stress in personal life that could impact nursing. Moreover, future studies are recommended to examine this relationship more in-depth, as the literature is scarce on such studies, and to examine if there is an influence from patient safety culture on job stress. Furthermore, studies could be extended to more healthcare professionals, which would be advantageous to improve data dependability and prevent confounding. In addition, nursing education should focus on training students on safety issues and conducting specialized safety classes, and emphasizing resiliency and trauma-informed care for nurses. Also, healthcare institutions should train their nurse leaders and nurses on how to manage and mitigate their stress.

## Supplementary Information

**Additional file 1. **PubMed search strategy.

## Data Availability

The data generated and analyzed in this study are included in the supplementary information file. Datasets are available through the first author upon reasonable request.

## Abbreviations

PSC: Patient Safety Culture
HSOPSC: Hospital Survey of Patient Safety Culture
JCIA: Joint Commission International Accreditation
PRISMA: Preferred Reporting Items for Systematic Reviews and Meta-Analyses
