# Supplementary material for: The relationship between job stress and patient safety culture among nurses: a systematic review
Source: BMC Nurs. 2023 Feb 13;22:39. doi: 10.1186/s12912-023-01198-9 (PMC9926568; doi:10.1186/s12912-023-01198-9)
Supplement: Supplementary file 1 — Additional file 1. PubMed search strategy. [file 12912_2023_1198_MOESM1_ESM.docx]

**Additional File 1**

**PubMed search strategy**

**#1** = occupational stress OR "work related stress" OR "job related stress" OR "organizational stress" OR "Job Stress" OR "professional stress" OR "workplace stress" OR "work stress"

**#2** = "patient safety culture" OR "safety culture" OR "patient safety" OR "safety climate"

**#3** = nurses OR nursing Staff OR registered nurses OR nursing personnel OR nurse*

**((#1) AND (#2)) AND (#3) =**

(("occupational stress"[MeSH Terms] OR ("occupational"[All Fields] AND "stress"[All Fields]) OR "occupational stress"[All Fields] OR "work related stress"[All Fields] OR "job related stress"[All Fields] OR "organizational stress"[All Fields] OR "Job Stress"[All Fields] OR "Professional stress"[All Fields] OR "workplace stress"[All Fields] OR "work stress"[All Fields]) AND ("Patient safety culture"[All Fields] OR "safety culture"[All Fields] OR "Patient safety"[All Fields] OR "Safety climate"[All Fields]) AND ("nurse s"[All Fields] OR "nurses"[MeSH Terms] OR "nurses"[All Fields] OR "nurse"[All Fields] OR "nurses s"[All Fields] OR ("nursing staff"[MeSH Terms] OR ("nursing"[All Fields] AND "staff"[All Fields]) OR "nursing staff"[All Fields]) OR ("nurses"[MeSH Terms] OR "nurses"[All Fields] OR ("registered"[All Fields] AND "nurses"[All Fields]) OR "registered nurses"[All Fields]) OR ("nursing staff"[MeSH Terms] OR ("nursing"[All Fields] AND "staff"[All Fields]) OR "nursing staff"[All Fields] OR ("nursing"[All Fields] AND "personnel"[All Fields]) OR "nursing personnel"[All Fields] OR "nurses"[MeSH Terms] OR "nurses"[All Fields] OR ("nursing"[All Fields] AND "personnel"[All Fields])) OR "nurse*"[All Fields]) AND ("2017/04/09 00:00":"3000/01/01 05:00"[Date - Publication] AND "english"[Language])) AND ((y_5[Filter]) AND (english[Filter]))
